# Virtual Inspection System for Pumping Stations with Multimodal Feedback

**DOI:** 10.3390/s24154932

**Published:** 2024-07-30

**Authors:** Zhiyu Shao, Tianyuan Liu, Jingwei Li, Hongru Tang

**Affiliations:** School of Electrical, Energy and Power Engineering, Yangzhou University, No. 88 South University Road, Yangzhou 225009, China; ltianyuan2022@163.com (T.L.); jingwei_li@yzu.edu.cn (J.L.); yztanghr@163.com (H.T.)

**Keywords:** virtual inspection, haptic force feedback, virtual reality (VR) simulator

## Abstract

Pumping stations have undergone significant modernization and digitalization in recent decades. However, traditional virtual inspections often prioritize the visual experience and fail to effectively represent the haptic physical properties of devices during inspections, resulting in poor immersion and interactivity. This paper presents a novel virtual inspection system for pumping stations, incorporating virtual reality interaction and haptic force feedback technology to enhance immersion and realism. The system leverages a 3D model, crafted in 3Ds Max, to provide immersive visualizations. Multimodal feedback is achieved through a combination of haptic force feedback provided by a haptic device and visual information delivered by a VR headset. The system’s data platform integrates with external databases using Unity3D to display relevant information. The system provides immersive 3D visualizations and realistic force feedback during simulated inspections. We compared this system to a traditional virtual inspection method that demonstrated statistically significant improvements in task completion rates and a reduction in failure rates when using the multimodal feedback approach. This innovative approach holds the potential to enhance inspection safety, efficiency, and effectiveness in the pumping station industry.

## 1. Introduction

Pumping station engineering in the water conservancy system serves numerous functions, including water supply, irrigation, and drainage, and has a significant impact on the management of water resources. However, pumping stations are typically situated in remote locations, making it challenging for workers to monitor and manage them effectively. As the modernization of the water conservancy system continues to advance, the inspection of pumping stations has become increasingly crucial in ensuring safety and preventing accidents.

Traditional pumping stations often face numerous challenges, including complex environments, low efficiency in line inspections, and difficulties in deploying personnel. These challenges stem from the extensive size of pumping station systems, the multitude of equipment, and complex inspection routes, leading to inefficiencies and high labor and resource costs. Furthermore, traditional inspection methods often require personnel to work with energized equipment, posing safety risks. The manual collection and recording of data is also time-consuming, prone to errors, and can lead to outdated information. Finally, training pumping station inspection personnel through traditional hands-on methods is time-consuming, costly, and can result in inconsistent training quality.

With the advancement of Computer Communication Technology, the installation of sensors and monitoring of operating parameters has made virtual inspection a reality. This not only reduces costs but also increases efficiency [1,2]. Virtual inspection, leveraging this technology, offers a compelling solution to these challenges. It significantly enhances inspection efficiency, reduces costs, eliminates safety hazards associated with live electrical equipment, streamlines data management, and improves the effectiveness and efficiency of operator training.

In the process of optimizing inspection systems and addressing related challenges, numerous research efforts have been made. Japanese scientists were the first to proposed a method to replace manual inspection in 1988. This system was capable of detecting map images and timely processing data in real-time [3]. Subsequently, American scientists also developed a similar inspection system [4]. By this point, the inspection system had gradually evolved from requiring personal to be physically present at the specified location to performing remote inspections. With further technological advancements, in 2010, the North American Hydroelectric Research Institute developed a patrol system called the Pacer, which was not limited to tracks and utilized video technology for remote control [5]. This marked a significant step in remote inspection technology. Recognizing the limitations of purely remote inspection methods, Srijeet Halder proposed a more advanced approach [6] using human–robot teaming. Halder compared interviews of professionals who completed the same inspection tasks twice: once using traditional methods and once using a robot. He found that these experts could rely on the robot for future construction inspection and monitoring if it were adopted. Although these inspection systems allowed for real-time observation of equipment status, they were still limited to remote inspections and had not yet entered the realm of virtual inspections.

During that time, the management of pumping stations in China was primarily in the basic stage of electrification, with intelligent management still in its early stages of exploration [7,8]. However, with the advancement of automation technology, the management techniques of pumping stations have seen significant improvement. In 2010, Wang Yue introduced a substation inspection system based on RFID technology [4]. This system aided operators in recording fault information for power equipment, addressing the challenge of personnel deployment difficulties in traditional methods. In 2018, Li Yahui designed an equipment operation and maintenance system that integrated mobile terminals and remote servers [9]. These designs for pumping station are gradually transitioning from traditional paper reports to electronic inspection systems [10]. Nevertheless, most existing inspection systems rely on equipment coding, infrared scanning, radio frequency identification, or other technologies. These systems often require chips or card readers, which add complexity and increase cost [11,12,13,14]. At this juncture, the concept of virtual inspection was proposed.

In 2020, Wu Di [15] proposed a wearable AR/MR device for human–machine interaction and remote information collection during virtual inspection processes. This innovation marked a significant milestone as it signaled the gradual transition of inspection towards true virtual inspection. The device enabled operators to seamlessly interact with machines, collect data remotely, and conduct inspections in a virtual environment, eliminating the need for physical presence at the inspection site. This not only enhanced safety but also improved efficiency, driving the immersion of operators in the process of virtual inspection, which has emerged as the primary research direction.

While VR has shown promise in the entire design process, from early conceptualization to detailed design and product evaluation, its benefits for engineering and product design have not been fully explored [16]. Khaled Aati’s research [17] focused on a VR-based inspection training system for the Missouri Department of Transportation. The immersive work zone proved effective, with an overwhelming majority (97%) of participants agreeing that VR provides a realistic and effective training method. This success demonstrates the potential of VR to enhance user efficiency by seamlessly integrating reality and virtual elements. Kamran Latif [18] developed a digital twin-driven framework for predicting Tunnel Boring Machine (TBM) performance. While this framework does not utilize VR technology, it establishes a vital link between the virtual and real world, integrating machine learning and real-time data for visualizing and monitoring tunnel construction progress. Combining this approach with VR technology could significantly minimize project risks and costs. In the complex aircraft maintenance industry, VR has emerged as a valuable training tool. Jeenal Vora [19] highlighted a VR system for aircraft inspection training that outperformed a traditional PC-based simulator, demonstrating its superior effectiveness.

Virtual inspection for pumping stations represents a significant technological leap forward, transitioning from physically demanding on-site inspections to digitally immersive remote control [20,21]. This shift enhances safety, convenience, and efficiency while improving accuracy. The virtual inspection system for pumping stations leverages immersive interaction based on virtual reality. VR, originally popularized in entertainment and gaming, has now found its way into industrial applications, leading to improved product quality, shorter development cycles, and cost savings [22,23]. Umair [24] observed that participants demonstrated better work performance in virtual environments, identifying more errors compared to traditional drawings. By immersing operators in a highly realistic environment, VR allows them to experience the pumping station as if they were physically present, even when working remotely. This immersive experience can reduce task completion times and improve professional efficiency. The virtual inspection system enables operators to remotely inspect and monitor pumping stations.

By employing VR in inspection systems, operators can experience a highly realistic and immersive environment, as if they were physically present at the pumping station, even when working remotely. This immersive experience can reduce task completion time and help professionals work more efficiently.

The construction of the virtual inspection system involves the use of computer graphics to create a three-dimensional model of the pumping station. Then, this model is rendered using an external VR device, providing operators with a highly realistic and interactive experience. Through VR control devices, operators can simulate the actions they would take on-site, such as pressing buttons or monitoring equipment.

In this step, the quality of the 3D model is a crucial factor influencing the user’s immersion. There are numerous methods for building 3D models, which we categorize into automatic and manual modeling approaches. Automatic modeling methods, such as UAV Oblique Photography Modeling Technology [25,26,27,28] and SFS (Shape from Shading) [29,30], involve the automatic generation of 3D virtual environments from 2D images. These methods are more efficient than manual methods but are more prone to errors. UAV technology was developed to enhance mapping abilities, initially in high-scale 3D/2D cartography and cultural heritage documentation. Later, it was applied in various fields with less stringent accuracy requirements, such as inspection. The pumping station company provided us with 3D models generated using UAV Oblique Photography Modeling Technology. Therefore, we did not participate in the entire 3D modeling process. While this method provides a general representation of the environment, it may not always meet the rigorous accuracy requirements for virtual inspection. Therefore, we employed manual modeling techniques to refine specific areas of the model where high accuracy was crucial—for example, the buttons on the electrical cabinet. This approach allowed us to combine the efficiency of automatic modeling with the precision of manual adjustments, resulting in a 3D model that balances fidelity and accuracy.

From rudimentary image recognition in early inspection systems to the use of wearable devices, the development journey demonstrates a significant leap in capabilities and immersion. Moreover, the introduction of haptic force feedback technology in virtual reality interactions represents a further leap in immersion. Haptic force feedback, which simulates the sense of touch, allows operators to feel vibrations, textures, and other physical sensations within the virtual world. This added layer of sensory input makes the virtual inspection experience even more realistic, enhancing the operator’s ability to identify and understand issues within the pumping station.

In 2002, Emst pointed out that visual deviation of human directly depends on the correction of tactile information [31]. When operators can receive haptic force feedback, they are better able to perceive and interpret the virtual world, leading to more accurate and efficient task completion. For instance, in comparative experiments involving manhole insertion operations, providing haptic force feedback to operators significantly reduced the time taken to complete the task compared to relying solely on visual cues [32].

The integration of haptic devices based on Tactile Reproduction Technology [33] into virtual inspection systems significantly enhances the operator’s experience and effectiveness. By providing haptic force feedback, these devices allow operators to interact with virtual objects in a more natural and intuitive manner, feeling their contours and tactile features [34,35,36]. This feedback loop enables operators to make adjustments and corrections to their actions in real-time, leading to more accurate and precise inspections [37,38,39]. Li Xinming pointed to a robot [40] that follows doctors’ natural operating habits and provides haptic feedback during surgeries. He believes that the application of this robot will reduce workload and shorten surgery time. Similarly, in virtual inspections of pumping stations, the application of haptic force feedback technology will also shorten inspection time and improve accuracy during inspections.

The traditional virtual inspection, which typically relies solely on visual cues, pales in comparison to the advanced virtual inspection system with haptic force feedback. The latter not only offers a more immersive experience but also enables operators to perceive and respond to changes in the virtual environment more naturally. Lee Jaeyeon proposed a system [41] where users can control microrobots remotely and feel haptic force feedback with the remote environment in real-time. This system ultimately improves the navigation performance and path tracking accuracy of the haptic operation for microrobot controls. In this study, the combination of VR technology and haptic force feedback technology creates a stronger sense of reality. This enhanced sense of reality makes it easier for operators to identify issues and potential problems within the inspected system, ultimately improving inspection performance and task accuracy during virtual inspections.

Currently, traditional virtual inspections of pumping stations prioritize visual experience and feedback over haptic feedback. While some virtual reality equipment (e.g., Oculus controllers) provide simple vibration feedback, it only conveys collision information. This equipment cannot effectively represent the haptic physical properties of devices during inspection, resulting in poor immersion and interactivity. In daily life, humans perceive physical properties through touch. Allowing users to touch virtual objects and perceive material properties, contours, and size would significantly bridge the gap between the virtual and real worlds [16,42]. Based on this, we incorporate force–haptic feedback devices to enhance immersion and operability.

Based on the above advantages, a virtual inspection system for pumping stations with multimodal feedback is established. Within this system, 3D scene models of the pumping station are generated, enabling operators to conduct inspections remotely using VR devices. Furthermore, haptic force feedback technology is incorporated, providing tactile feedback. This not only enhances the realism and immersion of the virtual environment but also allows operators to naturally perceive and respond to changes in the virtual space, ultimately leading to more precise and detailed operational insights. Additionally, the integration of IoT technology enables operators to access real-time data within the system, greatly assisting them in efficiently completing inspections.

This paper aims to investigate the impact of haptic force feedback on virtual inspection tasks for pumping stations. We begin by providing a comprehensive background review of virtual inspection techniques for pumping stations, the application of VR technology in industrial settings, and existing studies on haptic force feedback. Section 2 delves into the research methodology employed. Section 3 outlines the detailed building processes, which encompass three key steps: constructing a visual model, building a haptic model, and developing a data platform. Section 4 explains the application and testing of the system. The testing aims to assess the influence of haptic force feedback on operator performance by comparing the time taken and accuracy between a system with visual information only and a system with multimodal feedback (visual and haptic). Finally, Section 5 summarizes the study’s contributions and provides recommendations for future research directions.

## 2. Methods and Materials

**Figure 1:**
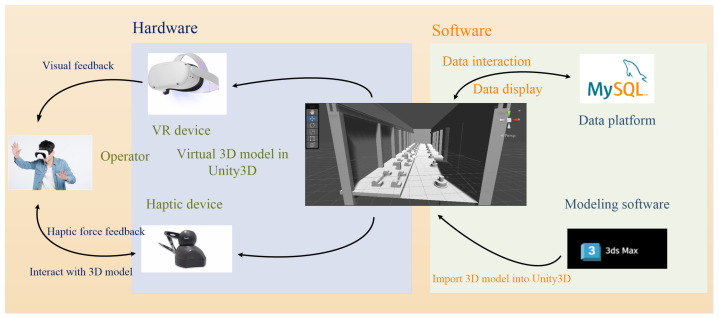
Structure of the inspection system.

This research builds upon the system depicted in Figure 1, with the primary objective of assisting operators in completing virtual inspections. This research aims to enhance the inspection process by combining the immersive capabilities of VR technology with haptic force feedback provided by haptic devices. Table 1 shows the main features of the VR headset and the haptic device.

Within this system, operators receive visual information via VR devices and haptic force feedback through haptic devices. Operators enter and explore the virtual world, interacting with virtual objects that are initially created in modeling software and subsequently imported into a rendering platform. Additionally, operators can access real-time data within the virtual world.

The system comprises three key components: a visual model, a haptic model, and a data platform. The visual model utilizes VR devices to provide operators with an immersive visual experience. The haptic model utilizes a haptic device to provide operators with haptic force feedback during virtual inspections. All data collected by the system are sent to the data platform, where they are managed and stored in a unified manner. When operators use the system, they can select and display specific data points from the platform.

### 2.1. Visual Model

This system employs VR devices to provide an immersive experience for operators during visual inspection. The Oculus Quest 2 (Meta Platforms, Inc., Menlo Park, CA, USA) VR headset serves as the primary interface for operators to access visual information. This headset, paired with VR controllers, forms the crucial visual information module of the VR virtual inspection system. This module is responsible for preparing and presenting visual information from the data platform in a way that fully utilizes the immersive capabilities of virtual reality.

Within the virtual environment, the visual information are integrated as 3D objects, providing operators with a realistic and engaging experience. The VR headset worn by the operator enables him to access and interact with all the information within the virtual world, from detailed representations of pumping station components to interactive data visualizations. This level of immersion not only makes the inspection process more intuitive but also improves the operator’s understanding of the system’s operational status.

To bridge the gap between these devices and the virtual 3D model, a foundational 3D rendering engine is essential. This engine facilitates the interaction and display of virtual objects, initially designed using specialized modeling software. This virtual inspection system leverages Unity3D (2021.3.29f1c1) as its core platform due to its comprehensive capabilities in real-time 3D rendering, physics simulation, and user interaction. Unity3D provides a robust programming environment supporting various languages like C# and JavaScript, enabling the development of interactive virtual experiences.

Context Capture generates 3D models from image sets using unmanned aerial vehicle (UAV) oblique photography modeling technology. This technology proves particularly efficient for rapid 3D reconstructions of real-world objects. The resulting models are then refined and optimized in 3Ds Max (20.0.0.966), a widely used 3D modeling software, before being imported into Unity3D.

### 2.2. Haptic Model

To enhance immersion and improve operator feedback during virtual inspections, we incorporated haptic force feedback into our system. The Geomagic Touch Haptic Device(3D Systems, Rock Hill, SC, USA) serves as the operator’s interface within the virtual world, providing tactile feedback. This device offers six degrees of freedom, allowing users to interact and manipulate 3D objects naturally, providing a tactile sense of the virtual environment.

The Geomagic Touch Haptic Device offers precise and responsive control, enabling operators to perform complex inspection tasks with ease. Its key specifications are detailed in Table 2.

We integrated the haptic device with the Unity3D platform to provide realistic force feedback. Our code implements a system that translates virtual object properties (e.g., material stiffness, texture) into haptic signals. This allows the device to provide force feedback based on the physical characteristics of the virtual objects, simulating a real-world interaction.

For example, when an operator interacts with a button within the virtual electrical cabinet using the haptic device, the code will generate appropriate forces depending on the button’s material properties. This allows the operator to “feel” the object’s material characteristics, enhancing the sense of realism.

### 2.3. Data Platform

Traditional inspection methods have become increasingly ineffective in the face of complex inspection problems, massive equipment, and vast amounts of data [43]. Our virtual inspection system, built upon a data modeling foundation, addresses this challenge by breaking down data silos, system islands, and management and control barriers. This approach transcends the limitations of traditional integrated systems, which often “monitor but do not control”. By integrating technology, data, equipment, and applications, our system enables the digitization, virtualization, real-time status tracking, and visualization of all building elements, providing rich operational data for informed sustainable development decision-making.

The data platform relies on IoT technology to remotely receive data from the pumping station. These data are then stored, managed, and modeled in a dedicated database. When operators utilize the inspection system, they can select specific data points from the database for analysis. Additionally, the system allows operators to modify certain data parameters within the database, enabling them to simulate different scenarios or test hypothetical changes to the system.

## 3. System Implementation

This study aims to establish a virtual inspection system with multimodal feedback, encompassing three key components: the visual model, the haptic model, and the data platform. The visual model serves as a 3D representation, utilizing UAV Oblique Photography Modeling Technology. Subsequently, any aberrations or inconsistencies are rectified in 3Ds Max, ultimately facilitating the importation of the virtual model into Unity3D. In terms of the haptic model, collision detection is initially conducted, followed by the classification of haptic forces into two categories: normal force and tangential force. Additionally, to enable operators to access real-time data within the inspection system, the establishment of a data platform is imperative. We leverage IoT technology to acquire data from the pumping station and subsequently store it in MySQL (8.0.13).

### 3.1. Building Visual Model

**Figure 2:**
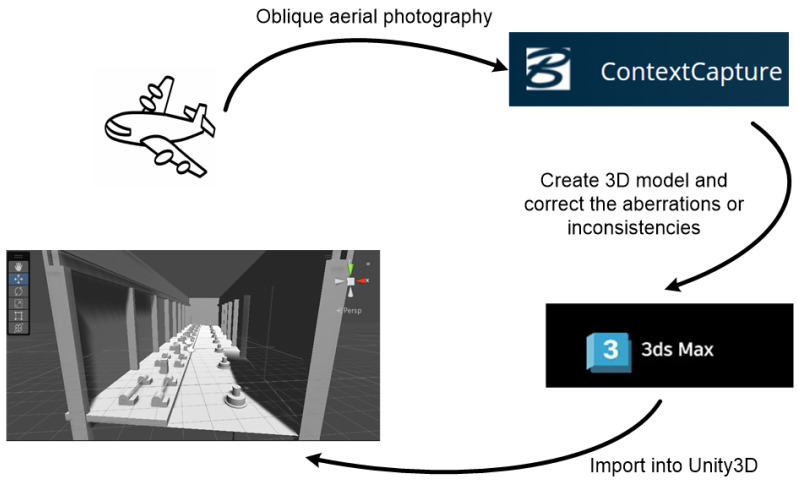
Process of building visual model.

The visual model for the system is constructed using UAV Oblique Photography Modeling Technology, as explained in Figure 2. The images and flight altitude information provided by the pumping station are used to build this model. This technology utilizes images captured by the UAV at low altitudes to create a highly accurate 3D representation of the target area. The sensor mounted on the UAV captures high-quality images [26] from various angles, enabling precise measurements [27] based on the UAV’s flight altitude information and integrated longitude and latitude coordinates.

To ensure the quality of the 3D model, the oblique photography images must meet specific technical criteria. Firstly, the images must contain recognizable datum points on the ground. These points serve as references for image orientation and point cloud positioning during the modeling process. Secondly, adjacent images must overlap to facilitate the generation of a seamless 3D model.

The captured images and flight altitude information are imported into the Context Capture Center System, which serves as the core of the modeling process. This system first generates a 3D mesh model without texture. Subsequently, it creates a textured 3D model by mapping the original images onto the mesh.

In this study, the pumping station company provided us with 3D models generated using UAV Oblique Photography Modeling Technology. Therefore, we did not participate in the entire 3D modeling process. However, due to limitations inherent in UAV Oblique Photography Modeling Technology, these models can exhibit inaccuracies, particularly for smaller objects. For instance, while the overall workshop housing the pumps might be represented accurately, smaller objects like electrical cabinets—and especially the buttons within them—are susceptible to distortion during the modeling process. Directly utilizing these distorted models would diminish the immersive experience during inspection. To address this, we manually modeled these smaller objects in 3Ds Max, ensuring their high accuracy. We then combined these manually created models with the automatically generated ones, making adjustments as needed.

Model accuracy verification can be approached from two perspectives: objective criteria (e.g., [44]) and subjective user feedback. While high accuracy under objective criteria is important, this study focuses on user experience, as a model’s effectiveness in this system is not solely determined by objective accuracy. User judgment, however, is subjective and prone to variability, leading to potential errors and requiring significant time. Therefore, this study employs a combined approach, incorporating both subjective and objective methods to verify model accuracy.

To assess the objective accuracy of the 3D models, we measured the coordinates of ten positions using RTK and compared them to the generated real scene model coordinates. This analysis met the requirements outlined in the Specifications for aerophotogrammetric office operation of 1:500 1:000 1:2000 topographic maps (GB/T 7930-2008) [44]; however, it is important to note that our primary focus was on achieving a recognizable virtual 3D model of the real object rather than strict metric accuracy. Finally, we conducted interviews with pumping station staff to gather subjective feedback on the accuracy of the integrated models. After approval, the final model was exported and imported into Unity3D for rendering the virtual 3D models.

### 3.2. Building Haptic Model

Haptic force feedback plays a crucial role in creating immersive experiences within virtual environments, particularly during interactions with virtual objects. This study designed a method for operators to receive haptic feedback. When operators use devices to interact with virtual objects, the system first performs collision detection. Upon a confirmed collision, the system generates haptic force feedback, enhancing the realism and immersion of the virtual inspection process.

#### 3.2.1. Collision Detection

In the virtual reality system, collisions between objects are essential for maintaining realism and believability. The timely detection and processing of these collisions, known as the collision process, is crucial for delivering a convincing virtual experience. This process consists of three key steps: collision detection, collision confirmation, and collision response.

Collision detection is the first step, which aims to determine whether two or more objects within the virtual environment are intersecting or overlapping. This is achieved by analyzing the geometric representations of the objects.

The second step is collision confirmation. Following detection, the system verifies if the colliding objects are designated as “touchable”. This decision relies on the properties and settings assigned to the virtual objects. For instance, certain objects may be defined as untouchable, preventing them from engaging in collisions even if overlap occurs.

The final step is collision response. Once a collision is detected and confirmed as touchable, the system determines the appropriate response. This typically involves providing haptic force feedback to the operator, which is provided by the haptic devices. To achieve this, the system needs the physical properties of the colliding objects. These properties, such as roughness, hardness, and viscosity, are mapped to the virtual objects. When a collision is confirmed, the relevant physical properties of the colliding objects are sent to the haptic devices. The haptic device then generates the appropriate haptic force feedback based on these properties, providing the operator with a realistic sensation of the collision.

#### 3.2.2. Building Haptic Force

**Figure 3:**
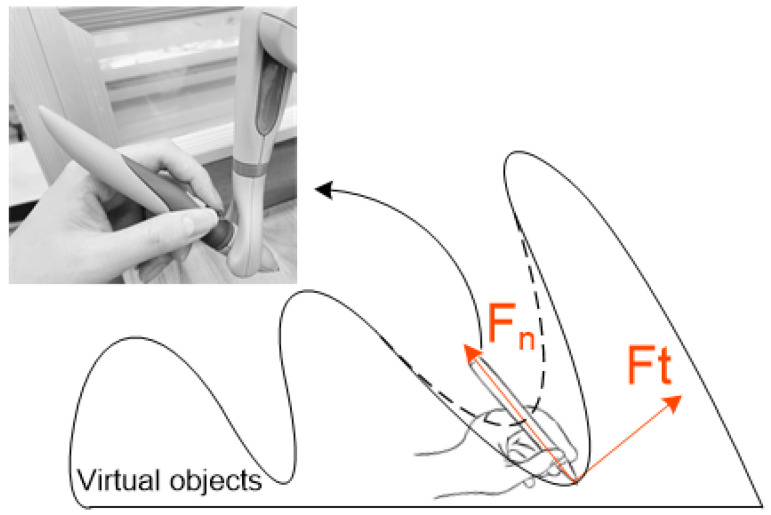
Depiction of haptic force feedback in virtual inspection system: normal force and tangential force.

We separate the haptic force feedback during collision into normal force ($F_{n}$) and tangential force ($F_{t}$). The normal force refers to the force that is perpendicular to the surface of an object, while the tangential force is the force that is parallel to the surface of the object. The haptic force feedback rendering is illustrated in Figure 3, and the specific mathematical expressions are as follows:

(1) $$F=F_{n}+F_{t},$$

According to the psychological effects of human interaction in virtual environments, when a haptic force device collides with a virtual object, the depth of the device’s virtual model entering the surface of the virtual object is directly proportional to the normal force ($F_{n}$). As shown in Equation (2), the coefficient *k* can be manually adjusted, and depth represents the depth at which the force device model penetrates the surface of the virtual object.

(2) Fn=k×depth,

The tangential force ($F_{t}$) includes the frictional force between objects, as shown in Equation (3), where μ is the coefficient of friction.

(3) Ft=μ×Fn,

### 3.3. Building Data Platform

The visualization of real-time data forms a critical aspect of virtual inspections, enabling operators to access and interpret live information within the virtual environment. To facilitate this, a robust data platform is essential. The development of such a platform begins with the construction of a comprehensive data model, which defines the structure and relationships of the collected data. Subsequently, the collected data are stored within a database for efficient management and retrieval.

#### 3.3.1. Data Preparation

After collaborating with pumping station staff and confirming site environment requirements, we determined that the system includes nine pumps. Each pump is equipped with multiple sensors to monitor various data points, such as hydraulic cylinder temperature and vibration amplitude. This necessitates a data preparation process. We confirmed that data are collected at a rate of every five minutes. Finally, we confirmed the specific data for visualization in the system.

#### 3.3.2. Building Data Model

The data collected in a pumping station can be categorized into two main types: master data and parameters. Master data refer to the basic information about the operating equipment, such as the equipment name, equipment code, equipment model, etc. Parameters are various technical indicators that represent the operational status of each equipment. These parameters can be categorized into direct and indirect parameters. Direct parameters are those that can be directly obtained, such as voltage and current. Indirect parameters are those that need to be calculated, such as water level and active power.

Based on the characteristics of pumping station data, this design aims to develop a pumping station data model. Since there are multiple data sources, it is necessary to integrate and store the data from various sources into a single database. Navicat, a database management tool, is used to import data into the MySQL database, a popular open-source relational database management system. We selected some important data for this inspection to store in the database.

#### 3.3.3. Data Collection and Transmission

**Figure 4:**
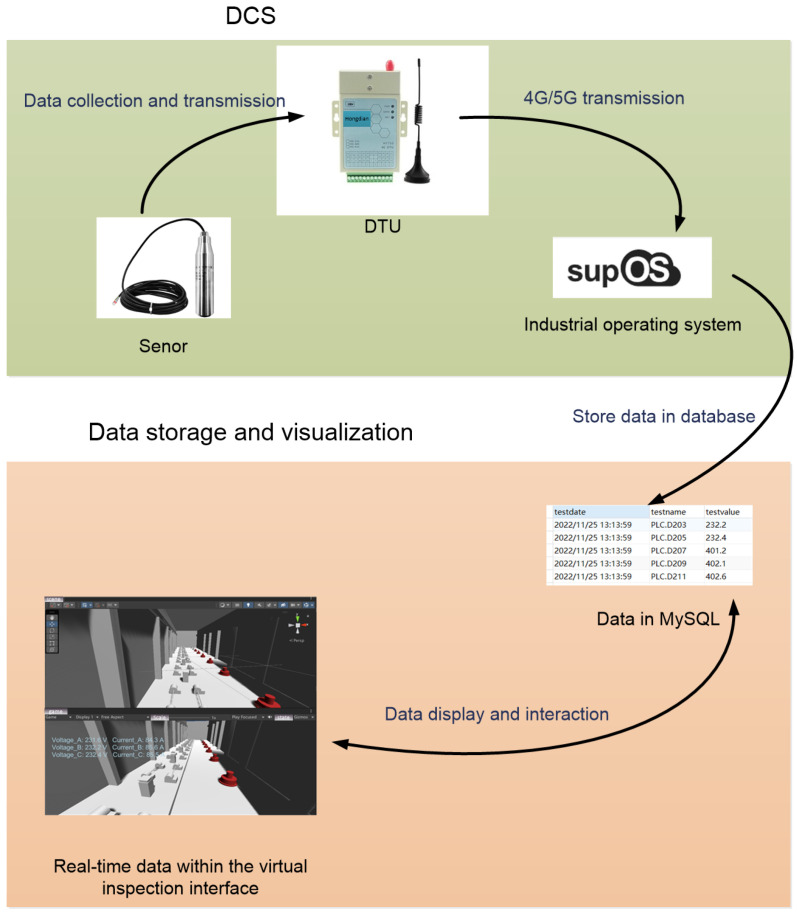
Process of data acquisition and transmission.

To enable real-time data utilization within intelligent management and virtual inspection systems for pumping stations, acquiring diverse information from the pumping station’s information management system is crucial. Establishing a unified data platform for seamless data collection and transmission is, therefore, an indispensable step. Figure 4 illustrates the process of data acquisition and transmission within this system.

Data acquisition primarily occurs through integration with a Distributed Control System (DCS), providing real-time process variables and equipment status information. These data are then stored within a designated database for further analysis and utilization. Initially, sensors deployed within the pumping station transmit collected data to IoT access devices such as Data Transfer Units (DTUs), which facilitate data transfer, and IoT gateways, which connect devices to the network. These devices then relay the sensor-acquired parameter information to the SupOS platform via network communication protocols. SupOS, an IoT operating system designed to simplify the development and management of connected devices, acts as a central hub, receiving and storing the collected data within the designated database. Users engaging with the pumping station’s virtual inspection system can readily access and query the stored data, which are then displayed in real-time within the virtual inspection interface.

#### 3.3.4. Data Management and Visualization

Data management is a crucial step in building a robust data platform. After collaborating with pumping station staff and confirming site environment requirements, we established a database to collect and store relevant data. Table 3 presents a summary of the data collected.

To cater to the specific needs of pumping station staff, we designed the virtual inspection interface to facilitate data selection and visualization. Operators can readily select and view specific data points of interest, such as current and voltage readings, through the interface. Additionally, the interface provides options for data visualization, such as graphical representations of water level height.

Furthermore, the data platform can perform computations on selected data points and store the results within the database. For instance, the system can calculate the “power-on time” by using the last power-on time and the last power-off time. This capability enables the system to provide valuable derived information for analysis and decision-making.

#### 3.3.5. System Design and Implementation

**Figure 5:**
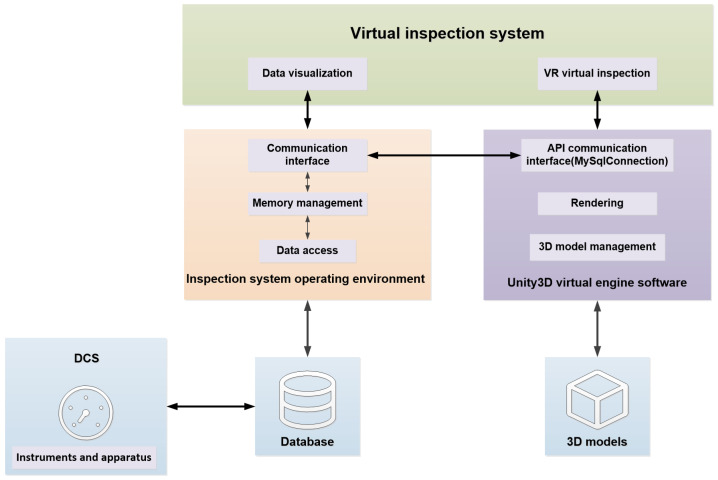
Data structure.

This project utilizes Unity3D, a full-featured 3D simulation engine. Unity3D offers an API interface, enabling its 3D virtual software to communicate with external systems. The software can display analog and device status within the virtual environment. For example, through interaction with the virtual control room model developed in this study, users can simulate equipment operation. The system can also output operation instructions to the 3D model. Additionally, it can communicate with databases in other management systems, facilitating data interaction between 3D models and other systems. The overall data structure of this study is shown as Figure 5.

When a user interacts with an operable virtual object, the system receives operation instructions, sends the data to the 3D model, and updates the virtual scene to reflect the results. For response devices, the system retrieves their current state from the data and dynamically displays it.

## 4. Application and Testing

A pumping station in Jiangsu China, at the Taizhou Yinjiang Canal Administration of Jiangsu Province, is selected as the application case. This pumping station plays a critical role in transferring water from the Taizhou Yinjiang Canal as a vital component of the east route of China’s South-to-North water Diversion project and a strategic resource for Jiangsu’s coastal development. Existing inspection methods at this pumping station are time-consuming and labor-intensive, highlighting the need for a more efficient and cost-effective approach. To address these challenges, a novel virtual inspection system with multimodal feedback has been designed and implemented. This research aims to evaluate the effectiveness of this system in improving operational efficiency and reducing management costs at the pumping station.

### 4.1. Establishment of Virtual Inspection System

**Figure 6:**
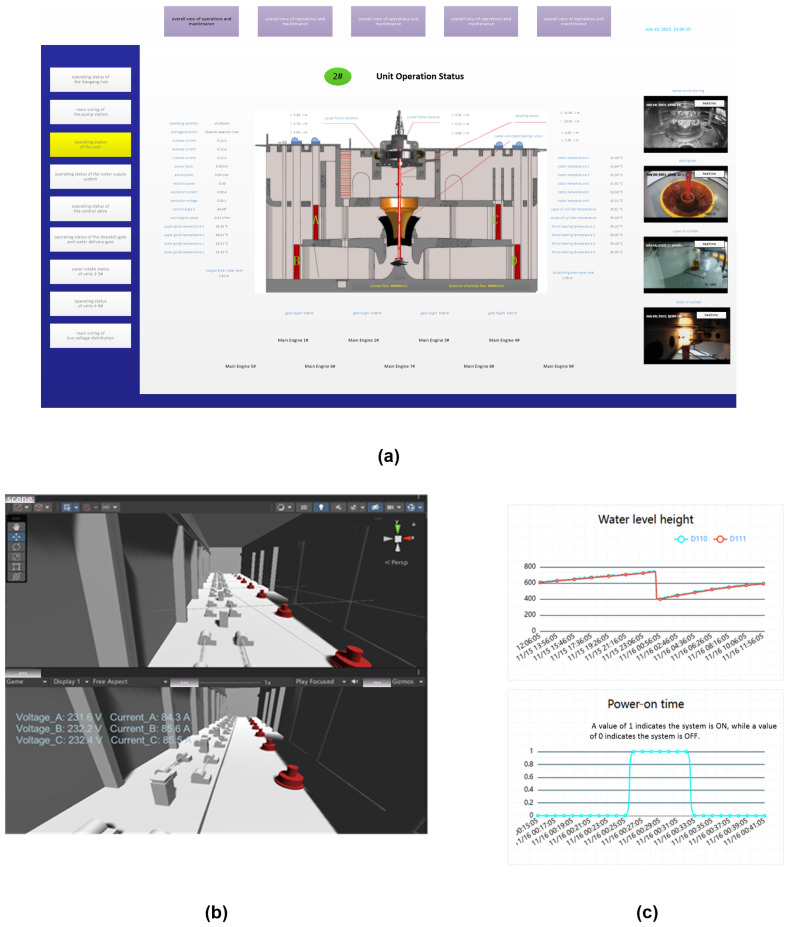
Comparison of system interface from traditional monitor system vs. virtual inspection system: (**a**) traditional monitor system; (**b**) virtual inspection system; (**c**) data visualization.

In 3Ds Max, we have revised the initial model provided by the pump station, which originally utilized the UAV oblique photography modeling technology. This model can be imported into Unity3D, where it is integrated with VR devices through C# scripting. Using the real physical surface information, when the haptic devices collide with the objects in virtual space, they can imitate haptic force feedback for operators. Figure 6 shows the comparison of the system interface from the traditional monitor system with virtual inspection system. Figure 6b shows the operator interface of the virtual inspection system in Unity3D. The traditional system is shown in Figure 6a. Figure 6c shows the data visualization.

### 4.2. Establishment of Data Platform

Real-time data display within the virtual inspection system is another crucial aspect of this application. To achieve this, a data collection and transmission process was implemented. Taking the reading of dyke water levels as an example, we will illustrate the process of data transmission and collection. Vibrating string-type water level gauges were chosen as sensors and deployed within the dyke structure. These sensors respond to changes in water pressure by altering electric current, providing data on water levels. This information is collected and transmitted to a DTU, which then sends the data to supOS, an industrial operating system responsible for data computation and storage within a database. This process forms the foundation of the designed DCS for the Yinjiang Canal Administration.

The virtual inspection system is designed to display and interact with the collected data. During operation, the system selects and displays appropriate data within the virtual inspection interface. Additionally, operator interactions with virtual objects within the system trigger updates to the database, ensuring a dynamic and responsive experience.

### 4.3. Validation

The following sections will use the designed virtual inspection system with multimodal feedback as an example to specifically validate the differences between this system and traditional virtual inspection systems. This validation will encompass both the collection of subjective results and a comparative analysis of the observed discrepancies.

#### 4.3.1. Experiment Aims

The aim of this experiment is to demonstrate that the proposed and designed virtual inspection system with multimodal feedback exhibits significant differences in accuracy and efficiency compared to traditional virtual inspection systems that rely solely on visual feedback. Since the difference between the two methods relies heavily on the operators’ reactions, it is challenging to gather objective evidence of the sense of reality. Unlike other objective metrics, subjective results are inherently difficult to assess. Therefore, psychological physics has frequently been used.

Taking into consideration the research subjects and the actual laboratory conditions, this study employs task-based psychophysical methods to collect data on inter-sample differences. Specifically, we will collect data on the time taken and accuracy achieved by subjects while performing the same task to analyze the presence of any significant discrepancies.

#### 4.3.2. Experiment Subjects

Eight participants (five male, three female) from Yangzhou University participated in the experiment. Their mean age was 24.2 ± 1.3 years. All participants were right-handed and had no known tactile perception deficits. The group consisted of six postgraduate students with expertise in pumping station management and two experienced pumping station staff. All participants possessed sufficient inspection experience in pumping stations. To minimize potential bias, three participants without prior VR experience received 30 min of VR experience through games at least one day before the experiment. All participants were blinded to the experiment’s objective. Before commencing, all subjects read the experimental instructions, signed informed consent forms, and received compensation for their time.

#### 4.3.3. Experiment Methods and Procedures

**Figure 7:**
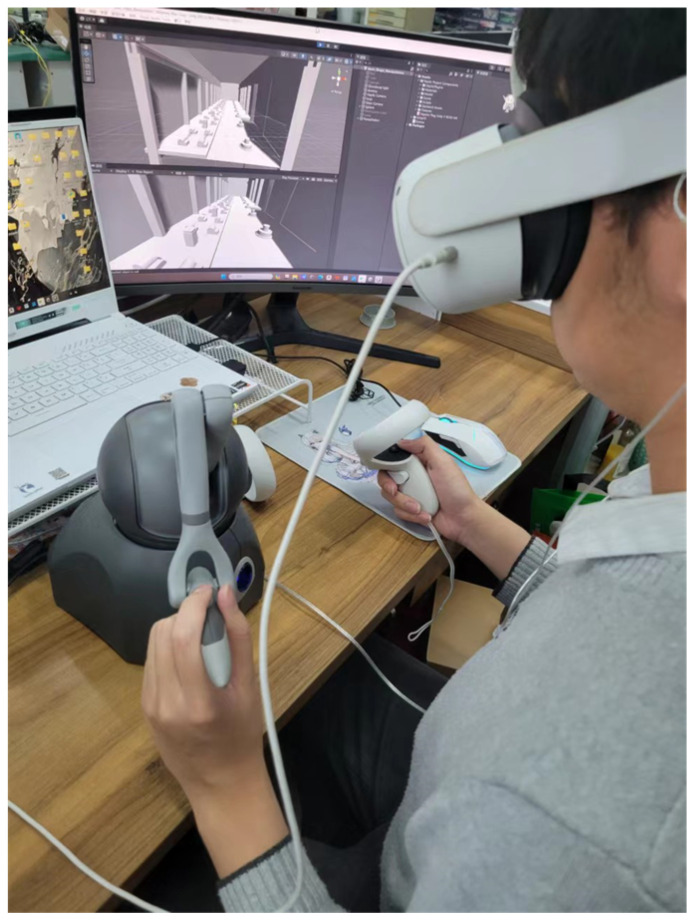
Operator is using the virtual inspection system.

This experiment employed task-based psychophysical methods to analyze subjective differences between the various systems under investigation. As shown in Figure 7, subjects were seated at a table, wearing a VR headset, and using a haptic device to interact with virtual objects within the virtual environment. We recorded subjects’ performance, including the time taken and accuracy achieved in completing the designated tasks.

The experiment was divided into two phases: a pre-experiment and a formal experiment. In the pre-experiment phase, subjects were introduced to the two virtual inspection systems and were given 3 min to familiarize themselves with the interaction methods using the haptic device and virtual objects. In the formal experiment phase, subjects were instructed to complete all tasks using both systems within 5 min. The order of the two systems was randomized for each subject. If a subject failed to complete the task within the allotted time, the trial was recorded as unsuccessful, and the time taken and accuracy metrics were recorded.

**Figure 8:**
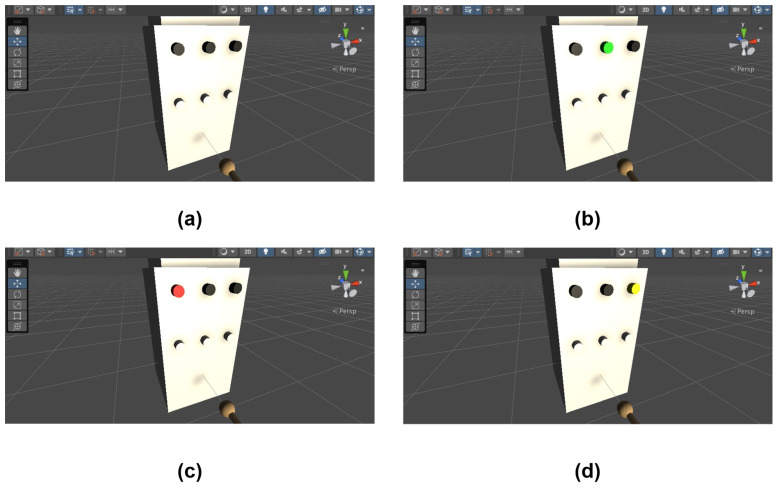
States of the virtual electrical cabinet. (**a**) Initial state. (**b**–**d**) Light-on states under various conditions.

In the formal experiment phase, subjects experienced the virtual environment visually and haptically. They were instructed to press three buttons on the virtual electrical cabinet within 5 min, following a specific order to simulate an inspection process. The corresponding indicator light on the cabinet illuminated only when the haptic device model touched the button and the designated button on the haptic device was pressed simultaneously. If the subject pressed the designated button but the indicator light failed to illuminate, the task was deemed unsuccessful. Figure 8 shows the different states of the virtual electrical cabinet.

#### 4.3.4. Experiment Results

**Figure 9:**
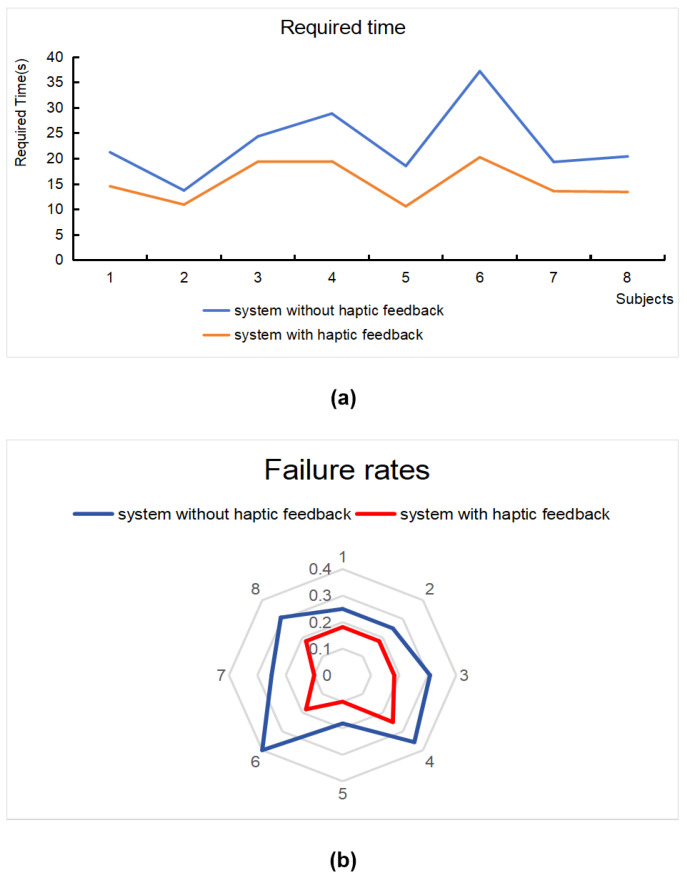
Comparison of required time and number of failures in different systems: (**a**) required time; (**b**) failure rates.

During the task-based experiment, subjects used a haptic device to interact with virtual objects, and their required time and failure rate within different virtual inspection systems were recorded. The required time and failure rate are shown in Figure 9, which demonstrates significant progress with the two inspection systems.

The required time is depicted in the line chart of Figure 9a. The haptic force feedback system showed less required time with an average of 15.32, while the system without haptic force feedback showed more required time with an average of 23.01. The failure rates of the two system are shown in the radial chart of Figure 9b. It is also shown that the system with haptic force feedback has a lower failure rate than the one without.

The findings suggest a difference in effectiveness and correctness between the two systems. Statistical analysis using a *t*-test (significance level of 0.05) confirmed these differences. The analysis revealed a significant difference in required time (*p* = 0.0193) and failure rates (*p* = 0.0015) between the two groups.

While it is possible that some participants might not benefit from haptic feedback, we did not observe any such outliers in our sample of eight subjects. However, future investigations with a larger participant pool will be necessary to conclusively determine if this is a general trend or an isolated finding.

## 5. Conclusions and Discussion

### 5.1. Discussion

This study developed a novel virtual inspection system for pumping stations, incorporating both virtual reality interaction and haptic force feedback technology. Our findings demonstrate that the inclusion of multimodal feedback significantly enhances inspection performance compared to traditional methods and systems without haptic feedback. While the current study involved a limited number of test users, the results demonstrate a promising trend: the multimodal system significantly reduced average task completion time compared to the system without haptic feedback. Additionally, the failure rate was lower in the multimodal condition. Future research with a larger sample size is needed to confirm and further explore these findings. Nevertheless, the current results suggest that even with an expanded participant pool, the multimodal feedback system is likely to maintain its higher efficiency and lower failure rate compared to the traditional system.

This improvement in performance likely stems from the enhanced realism and immersion provided by the haptic feedback [34,35,36]. These results are consistent with previous research demonstrating the positive impact of haptic feedback on task performance in various domains [37,38,39]. For example, Lee Jaeyeon [41] proposed a system where users can control microrobots remotely and feel haptic force feedback with the remote environment in real-time; this enhanced sense of reality makes it easier for operators to identify issues and potential problems within the inspected system, ultimately improving inspection performance and task accuracy during virtual inspections. While Li Xinming [40] demonstrated its benefits in training for surgical procedures. Our findings further support the growing body of literature highlighting the potential of haptic feedback to enhance user experience and performance in VR applications.

The findings of this study have significant implications for the future of virtual inspection systems in the pumping station industry. The use of VR with haptic feedback offers a number of advantages over traditional inspection methods:

- Improved Efficiency and Accuracy: The reduced task completion time and lower failure rate observed in this study suggest that the virtual inspection system with haptic feedback can lead to substantial improvements in efficiency and accuracy.
- Enhanced Safety: VR-based inspection systems can provide a safe and controlled environment for training and practicing inspection procedures, reducing the risk of accidents and injuries associated with real-world inspections.
- Reduced Costs: The virtual inspection systems with multimodal feedback can potentially reduce the need for costly and time-consuming on-site inspections, leading to significant cost savings.

While promising, it is important to acknowledge the limitations of this study. VR users can experience motion sickness, often referred to as VR sickness or cybersickness [45]. While the experimental subjects were selected based on their knowledge of pumping stations or experience in managing them, expanding the subject pool is a priority for future research. In addition, evaluating participants’ cognitive load (CL), task performance (TP), and situational awareness (SA) in the VR environment is crucial [24]. Future research and testing will focus on exploring these aspects, particularly in more complex and realistic interaction scenes.

Furthermore, the current prototype focused on specific haptic feedback cues related to surface properties: hardness, roughness, and stickiness. Future research should explore a wider range of haptic feedback modalities, including those related to the shape and quality of virtual objects. Specifically, the development of haptic gloves or other advanced haptic devices could greatly enhance the realism and immersion of the VR experience. Additionally, research on the integration of visual and tactile feedback in VR environments holds significant promise for improving user understanding and performance in complex inspection tasks.

The findings of this study provide compelling evidence for the potential of VR-based inspection systems with haptic feedback to significantly enhance inspection performance in the pumping station industry. Future research should focus on addressing the limitations of this study and exploring the full potential of this technology for improving efficiency, accuracy, safety, and cost-effectiveness in industrial inspection applications.

### 5.2. Conclusions

This study underscores the potential of VR-based virtual inspection systems with haptic feedback for enhancing inspection efficiency and accuracy in pumping stations. While the current study involved a limited number of test users, the findings suggest a promising trend: incorporating haptic feedback into the virtual inspection process led to a notable reduction in the average task completion time compared to the system without haptic feedback. This suggests a potential for improvement in operator performance. These results underscore the positive impact of haptic technology on realism, immersion, and potentially operator effectiveness in virtual environments. This research suggests that the inspection system with multimodal feedback enhances users’ understanding of cognitively demanding problems, thereby potentially improving their efficiency in virtual environments. Increased inspection efficiency, driven by both faster completion times and potentially enhanced accuracy, can lead to significant cost savings and improved operational reliability for pumping stations and other industrial settings where inspections are critical. This technology holds particular promise for tasks involving complex equipment or hazardous environments. Future research with a larger sample size will be necessary to further confirm and quantify these potential benefits.

The limitations of this study lie primarily in the subject pool. Future research should expand the subject pool and refine participant selection by considering a broader range of factors, including age and experience related to pumping stations. While the current results suggest that the multimodal feedback system is likely to maintain its higher efficiency and lower failure rate even with a larger and more diverse participant pool, it is important to acknowledge the limitations of the current sample. Additionally, the current interaction scene is relatively simple, and the inspection time is short. This may explain why all participants perceived significant benefits from using the system. Future research should explore more complex and realistic interaction scenes and tasks, which may reveal more nuanced findings. Lastly, the current prototype focused on specific haptic feedback cues related to surface properties: hardness, roughness, and stickiness. These cues were simulated by adjusting the resistance force feedback of the haptic device. Future research should explore a wider range of haptic feedback modalities, including those related to the shape and quality of virtual objects, to further enhance the realism and effectiveness of virtual inspection systems.

Currently, the Taizhou Yinjiang Canal Administration has implemented this designed system. The primary technological components of this study include the accuracy of 3D model building, the immersion of haptic force feedback, and the real-time accuracy of data interaction and modeling. As 3D modeling technology advances, this system will become more interactive. However, the system’s immersion is heavily reliant on the development of augmented reality devices and technologies. More importantly, research into haptic force feedback models is critical, especially for different types of haptic devices (e.g., haptic gloves). Realizing the hardness and roughness of objects in a virtual environment is a key objective. Furthermore, the fusion mechanism of information processing resulting from the interaction of visual and tactile senses presents a significant research area.

## Figures and Tables

**Table 1:** Main features of the VR headset and the haptic device.

VR Headset	Haptic Device
Receive visual information	Receive haptic force feedback
Platform for 3D model and data visualization display	Generates different haptic force feedback depending on different physical factors

**Table 2:** Technical specifications of haptic device.

Device Specifications	Metric
Force Feedback Workspace	431 W × 348 H × 165 D mm
Backdrive Friction	<0.26 N
Maximum Exertable Force (at nominal orthogonal arms position)	3.3 N
Stiffness	X axis > 1.26 N/mm; Y axis > 2.31 N/mm; Z axis > 1.02 N/mm

**Table 3:** Stored specific data in MySQL.

Data Name	Data Type
Running state	Boolean
Rack X-direction vibration amplitude	Float
Rack Y-direction vibration amplitude	Float
Rack Z-direction vibration amplitude	Float
Current in phase A	Float
Current in phase B	Float
Current in phase C	Float
Voltage in phase A	Float
Voltage in phase B	Float
Voltage in phase C	Float
Active Power	Float
Reactive Power	Float
Power Factor	Float
Stator Temperature	Float
Hydraulic Cylinder Temperature	Float
Last power-on time	string
Last power-off time	string
Power-on time	string
Water level height (D110)	Float
Water level height (D111)	Float

## Data Availability

The datasets generated during and/or analyzed during the current study are available from the corresponding author on reasonable request.
